# Matching livestock to landscape: a proposal of metrics to phenotype grazing distribution from Global Navigation Satellite System collar data

**DOI:** 10.1093/af/vfad051

**Published:** 2023-10-13

**Authors:** Francisco Maroto-Molina, Eseró Padrón-Tejera, Dolores C Pérez-Marín

**Affiliations:** Department of Animal Production, School of Agricultural and Forestry Engineering, University of Cordoba, Cordoba, Spain; Department of Animal Production, School of Agricultural and Forestry Engineering, University of Cordoba, Cordoba, Spain; Department of Animal Production, School of Agricultural and Forestry Engineering, University of Cordoba, Cordoba, Spain

**Keywords:** animal breeding, climate change, grassland, precision livestock farming, rangeland

**Implications**

- Spatial distribution is a critical component of climate-adapted grazing management. Traditional practices have not considered spatial patterns because they are difficult to measure and control. The availability of commercial Global Navigation Satellite System collars poses an opportunity to improve farmer decision-making by tracking livestock distribution.
- Researchers have used diverse metrics to phenotype grazing distribution. Most common indicators are slope use and distance to water, which are external drivers of livestock distribution, but not direct measures of grazing evenness.
- A transdisciplinary approach consisting of the adoption of evenness metrics typically used in ecology or economics may benefit grassland science and management. For the first time, the Lorenz curve and a Gini-type coefficient are proposed to describe grazing evenness. Potential applications of such metrics and further research needs are reported.

## Introduction

Grazing lands cover above one-third of Earth’s ice-free terrestrial surface, playing a key role as suppliers of ecosystem services, such as livestock feed production, water supply, erosion control, carbon storage, biodiversity conservation, etc. Since grasslands are reliant on weather, primarily rainfall and temperature, climate variability largely affects their performance.

Many extensive livestock farms are situated in arid and semi-arid areas, making them especially vulnerable to the impacts of climate change. In arid regions, increasing temperatures and declining rainfall are already being observed, and a shift in the seasonal distribution of pasture production (e.g., shorter growing seasons), as well as an increasing year-to-year climate variability, are also expected. Changing climate will require farmers to adapt their management practices to be sustainable and resilient.

Over the last few years, numerous adaptation strategies have been proposed, such as changes in species or breeds of livestock and fine-tuning of grazing practices (Joyce et al., 2013). According to Vallentine (2001), grazing management relies on four principles: 1) type of animal, 2) stocking rate, 3) timing of grazing, and 4) grazing distribution. Traditional grazing management has been based on choosing a species (frst principle) and optimizing stocking rate (second principle). To a lesser extent, the temporal dynamics of herbage allowance and demand (third principle) have also been considered. However, the spatial heterogeneity of grazing (fourth principle) has not received such attention, probably because it is harder to measure and control. Grazing animals tend to congregate in some areas within paddocks, leading to overgrazing, while others receive little attention. Optimizing livestock distribution may help to alleviate some effects of climate change, such as reduced pasture availability, through encouraging animals to graze areas that they normally avoid.

Technological advancement, in particular the availability of Global Navigation Satellite System (GNSS) collars aimed at commercial farms, is a paradigm shift regarding the possibilities of measuring and managing spatial grazing patterns. In this perspectives article, we review the main metrics of livestock grazing distribution reported in scientific literature and propose a new framework based on the Lorenz partial order and a Gini-type coefficient, which is aimed at facilitating the visualization and quantification of grazing evenness.

## Metrics of Livestock Grazing Distribution in Scientific Literature

Description and manipulation of spatial patterns of livestock have been broadly addressed by researchers from Western North America and Australia (Nyamuryekung’e et al., 2022). Topography and water location are main drivers of livestock site use preference, although many other factors can influence livestock concentration in certain areas (shade, mineral supplementation, pasture availability and quality, genetics, social structure of the herd, etc.). Strategies to modify animal grazing distribution include herding, fencing, water or supplement placement, and behavior conditioning, but most of them are expensive or difficult to implement. A strategy that avoids costly and labor-prohibitive approaches is the selection of breeds or individuals within a herd that exhibits desirable spatial patterns. According to Bailey et al. (2015), grazing distribution is a heritable trait, so it can be subjected to selection.

Many studies including metrics of grazing distribution built from GNSS collar data are targeted at cattle selection. The most commonly used metrics are stepper slope use and maximum distance to water. Bailey et al. (2015) built terrain use indices based on normalized averages of elevation, slope use, and distance traveled from water, which were used as phenotypes of grazing distribution to be associated with quantitative trait loci. Other authors have reported diverse indicators of grazing distribution: level of activity, time grazing, daily distance traveled, travel velocity, path sinuosity, home range (area explored), herd cohesion, selectivity of greenest patches, number of hotspots (areas of reuse), etc. (McIntosh et al. 2023). These indicators mostly refer to preconditions for less patchy grazing patterns, but they do not directly measure grazing evenness. Pauler et al. (2020) found some contradictory results on this. In their study, Highland cattle moved farther away from water than Braunvieh but, interestingly, they took fewer steps, covered less distance, and spent more time lying. Thus, the correlation between some of the indicators and grazing evenness might be not as straightforward as presumed.

It is worth mentioning that, in many cases, using indirect indicators may be perfectly suited to the objectives of the study. For example, Bailey et al. (2015), when studying cattle behavior on large ranches, assumed that most cows would not graze farther than 2 km from water sources. Thus, by selecting the animals traveling the largest distances from water, they were aiming at transferring grazing pressure from terrain near water to other areas, which might result in a more even spatial pattern of the herd. In the case of smaller grazing paddocks, such as those used in European countries, which rarely exceed 100 ha, there might be no terrain further than 2 km from water, but livestock grazing distribution is still not even. For those cases, the computation of evenness indices from GNSS collar data may be useful to enhance decision-making. According to our review, few studies in the field of grassland research have used evenness indices. As an example, Pauler et al. (2020) estimated grazing evenness through Camargo index (E’), which indicates the relative abundance of animal locations along farm sites.


$$E^{\prime }=1-[\underset{i=1}{\overset{s}{\sum^{\text{​}}}}\;\underset{j=i+1}{\overset{s}{\sum^{\text{​}}}}\;(\frac{\left|p_{i}-pj\right|}{s})]$$

where *p*_*i*_ is the proportion of animal locations at site *i*, *p*_*j*_ is the proportion at site *j* and *s* is the total number of sites, which might be grid pixels.

The use of evenness indices is, however, quite common in other scientific disciplines, especially in economics (income distribution) and ecology (biodiversity studies). Adapting these indices to describe livestock distribution may provide new tools for grassland farmers and researchers.

## Proposal of Lorenz Curve and Gini-Type Coefficient to Phenotype Grazing Evenness

Gosselin (2001) compared the performance of multiple evenness indices used in biodiversity studies, concluding that the best way to quantify the notion of evenness is the use of Lorenz partial order and evenness indices that are compatible with it, such as Gini coefficient (Camargo index is not fully compatible). Lorenz partial order was developed in economics, and, to the best of our best knowledge, it has not been previously used to describe livestock distribution.

**Figure 1.**
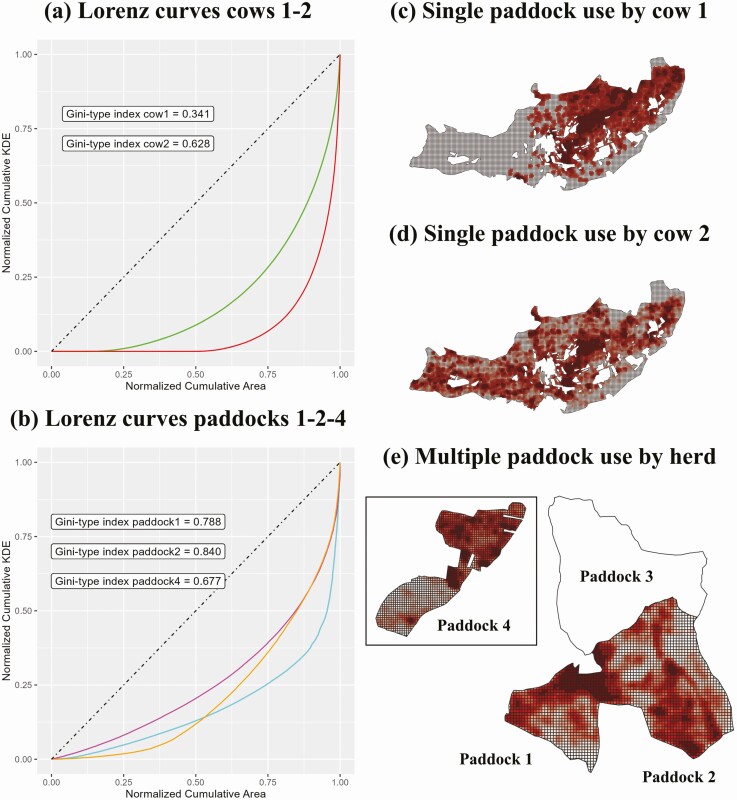
Lorenz curves and maps of paddock use for comparison of two individual cows (a, c, and d) and three paddocks grazed by the same herd (b and e).

In order to illustrate the potential of Lorenz curves and Gini coefficients for grassland management, two case studies are presented. A total of 78 cows were fitted with commercial GNSS collars (Digitanimal Ltd.) in two extensive farms located in Avila (central Spain) throughout the year 2021. The first farm consisted of a single grazing paddock with an area of 629 ha. Slopes above 20% were present in 46% of paddock area, while only 18% of the land could be considered flat (slope below 10%). The second farm had 338 ha divided into four fenced paddocks (43 to 108 ha). Maximum slope in all paddocks was approximately 20%, but the proportion of area with slopes above 10% was higher in paddock 2 (43%). Paddocks 1 and 4 were mostly flat terrain. Natural pastures were present on both farms. The most abundant genera were *Festuca*, *Koeleria*, *Poa*, *Agropyron*, *Dorycnium*, *Coronilla*, *Anthylis*, *Argyrolobium,* and *Hippocresis*. Some shrubs (*Cytisus*, *Thymus,* and *Crataegus*) and trees (*Quercus*) were also present in all plots. Collars were configured to provide location fixes for each cow every 30 min. We used cattle positions to compute kernel density estimates (kde) on a 25 × 25-m grid. Since location fixes were recorded at uniform time intervals, both the number of cow positions and kde values per pixel can be seen as indicators of the time that cattle spent in such pixels. Thus, kde data were ordered from lowest to highest and their cumulative sum was calculated and normalized. In parallel, the corresponding cumulative sum of pixel areas was also computed and normalized. We plotted the cumulative sum of areas (abscissa axis) against the cumulative sum of kde values (ordinate axis), obtaining the Lorenz curves shown in Figure 1(a-b).

If livestock distribution was completely even, i.e., the amount of time cattle spent at each pixel of the grid was the same, Lorenz curve would correspond to the diagonal of the graph. Thus, any curve below that diagonal line represents an uneven livestock distribution. Lorenz curves provide an easy way to compare grazing evenness for two or three animals, paddocks, farms, etc., since any curve above another represents a more even distribution. When it comes to the quantitative comparison of a higher number of entities, the use of a single value (evenness index) is more convenient. This is the role of Gini coefficient, which is the area between the Lorenz curve and the diagonal line, expressed as a proportion of the triangular area between the diagonal line and abscissas axis. Thus, the closer to zero the value of Gini (G), the greater the evenness of grazing distribution. This is counterintuitive when describing grazing evenness, as most indices used in ecological studies range from 0 (patchy) to 1 (even). For that reason, we propose using 1 – G as evenness index of livestock distribution.

In Figure 1a we plotted the Lorenz curves of the two cows with the highest and lowest Gini-type values for the first farm, revealing that phenotypic variability exists for this trait. Cow 1 (red line) had a more uneven distribution than cow 2 (green line), which is also clear in maps of paddock use (Figure 1c-d). Lorenz curves provide more insights into cow behavior than simple comparison. For example, it is easy to interpret that cow 1 did not use more than 50% of available land (0% of time), while cow 2 only avoided less than 25%. With Figure 1b we aimed at demonstrating the use of Lorenz curves to compare grazing evenness in several paddocks supporting the same herd. When comparing three of the four paddocks of the second farm, paddock 2 showed a more even grazing pattern than paddocks 1 and 4, since its Lorenz curve is situated everywhere above the other two curves. However, Lorenz curves of paddocks 1 and 4 intersect, which constitutes a great example of the complexity of grazing systems. Globally, it could be considered that paddock 1 (cyan line) was used more evenly than paddock 4 (orange line) because its Gini-type index is higher. Lorenz curves provide us additional insights. Half the land less used by livestock in paddock 1 showed nonetheless a more even grazing pattern than the correspondent half-less-used terrain of paddock 4. The opposite occurred in the most used half of the land, where grazing evenness was superior in paddock 4 (see Figure 1e). This information may serve to implement different strategies aimed at improving livestock distribution at each half-paddock.

## Conclusions

The optimization of livestock distribution may be an effective climate change adaptation strategy, but requires technologies, metrics, and tools that allow the description, manipulation, and tracking of spatial patterns of grazing animals. A new approach to the study and quantification of grazing distribution has been proposed in this perspectives paper. Through case studies, we demonstrated the potential of Lorenz curve and Gini-type coefficients to interpret the complexity of animal-landscape interactions. Our proposal complements grazing distribution indicators used in previous studies and deserves further investigation, e.g., on the effect of different parameters, such as grid size, on Gini-type values, or on the use of Lorenz curves to compare grazing evenness before and after modifying some management practices.

## About the Author(s)

**Francisco Maroto-Molina** is an Assistant Professor at the University of Cordoba, Spain. He holds an Agricultural Engineering degree and a PhD in Animal Science. His research focuses on the development and validation of precision technologies to support decision-making of extensive livestock farmers. He is currently working on the automatic detection of calving and estrus in beef cattle, sheep, and horses, as well as on the optimization of grazing through a better understanding of the interactions between animals and landscape. He is vice president of the Study Commission on Precision Livestock Farming of the European Federation of Animal Science and coordinator of Precision Livestock Farming courses for the Digital Competence Center of the Spanish Ministry of Agriculture, Fisheries and Food.

**Eseró Padrón-Tejera** is a PhD candidate at the University of Cordoba, Spain. He holds an Agricultural Science degree from the University of La Laguna, Canary Islands. He has studied a double master’s degree on Agricultural Engineering and Rural Development. His PhD thesis focuses on the effects of livestock grazing distribution on soil and pasture characteristics. He is also investigating the spatial dispersion of cattle feces on rangelands and its role in nutrient recycling.

**Dolores C. Pérez-Marín** is a Full Professor at the University of Cordoba, Spain. She holds a PhD in Agricultural Engineering. She has developed a successful research career in the field of nondestructive spectral sensors, addressing both basic, and applied research in a wide range of agrifood applications, with a special focus on animal feeds. She has developed extensive experience in multivariate modeling with large datasets. She is currently working on the use of portable low-cost NIRS sensors to characterize the spatial variability of soil and pasture characteristics on rangelands. She is president-elect of the International Council on Near Infrared Spectroscopy and coordinator of SensorFINT European researcher network.
